# The Role of Gut-Microbiota in the Pathophysiology and Therapy of Irritable Bowel Syndrome: A Systematic Review

**DOI:** 10.7759/cureus.28064

**Published:** 2022-08-16

**Authors:** Bijay Shrestha, Deepkumar Patel, Hriday Shah, Kerollos S Hanna, Harkirat Kaur, Mohammad S Alazzeh, Abhay Thandavaram, Aneeta Channar, Ansh Purohit, Sathish Venugopal

**Affiliations:** 1 Family Medicine, California Institute of Behavioral Neurosciences & Psychology, Fairfield, USA; 2 Neurology, California Institute of Behavioral Neurosciences & Psychology, Fairfield, USA; 3 Internal Medicine, California Institute of Behavioral Neurosciences & Psychology, Fairfield, USA; 4 General Practice, California Institute of Behavioral Neurosciences & Psychology, Fairfield, USA; 5 Orthopaedic Surgery, California Institute of Behavioral Neurosciences & Psychology, Fairfield, USA; 6 Research, California Institute of Behavioral Neurosciences & Psychology, Fairfield, USA

**Keywords:** visceral hypersensitivity, gut-brain axis, gut microbiota, ibs, irritable bowel syndrome

## Abstract

Irritable Bowel Syndrome (IBS) is one of the most prevalent chronic gastrointestinal diseases, which is characterized by recurrent abdominal pain and altered bowel habits. The pathophysiological mechanisms are not completely clear for IBS, multiple factors such as genetic, psychosocial, environmental, visceral hypersensitivity, low-grade inflammation, gastrointestinal motility changes, food components, and intestinal microbiota are thought to play a role in the disease process of IBS. The rapid progression of recent microbiome research using advanced microbiological technologies has shed light on dysbiosis related to the pathophysiology of IBS. We used PubMed, PubMed Central, and Medline as our primary databases to search for articles using keywords and medical subject heading (MeSH) keywords on April 30, 2022, to render a total of 4062 articles. Then, a total of 10 articles were selected following a quality assessment. Despite the variable findings in different studies, most studies have concluded that IBS patients have a reduction in bacterial diversity and an increase in the temporal instability of the microbiota. IBS is known as a *stress disorder,* and the gut-microbiome-brain axis has been associated with the pathogenesis of the disease. Additionally, the potential of dietary manipulation of gut microbiota and the use of probiotics, prebiotics, and synbiotics in the treatment of IBS has been studied in recent years and shown promising results. We concluded that the gut microbiome plays a substantial role in the pathophysiology of IBS.

## Introduction and background

Irritable Bowel Syndrome (IBS) is a chronic gastrointestinal disease of unknown etiology, which is characterized by recurrent abdominal pain and altered bowel habits [1]. Based on the symptoms, IBS is further divided into four subtypes, namely diarrhea-predominant IBS (IBS-D), constipation-predominant IBS (IBS- C), or mixed IBS (IBS-M), and unclassified IBS [2,3]. According to reports, in the United States, IBS-M is the most common subtype accounting for 44% of total cases of IBS, and approximately 26% and 28% of patients are diagnosed with IBS-D and IBS-C, respectively [3].

The global prevalence of IBS is approximately 10%-15% making it one of the most prevalent gastrointestinal disorders [4]. Roughly (12%-14%) of the total primary care visits and 28% of the gastrointestinal referral accounts for IBS cases [1]. IBS is usually diagnosed in the younger population under 50, and the female: male ratio is 2:1 [5]. The health-related quality of life (QOL) is seen to be negatively affected in IBS patients. Patients are usually seen suffering from comorbidities such as anxiety, depression, fibromyalgia, migraine, headache, interstitial cystitis, and temporomandibular joint syndrome [3,4]. Even though the pathophysiological mechanisms are not completely clear for IBS, multiple factors such as genetic, psychosocial, environmental, visceral hypersensitivity, low-grade inflammation, gastrointestinal motility changes, food components, and intestinal microbiota are thought to play role in the disease process of IBS [1,5,6]. Of note, because of the heterogeneous characteristics of IBS and the variability in the progression of the disease, the management of IBS patients remains a challenge to physicians. There are no specific biomarkers, or laboratory tests available for the evaluation of IBS and the treatment is mainly directed towards the primary symptoms of the patient, which includes various pharmacotherapy, and the other approaches include only lifestyle modification and dietary changes [2,5,7].

Thus, the precise mechanism of IBS has still not been completely understood, but several researchers have drawn attention to dysbiosis of the gut microbiota as one of the potential causes of IBS [4]. Trillions of microorganisms reside in the gastrointestinal tract and maintain a mutual relationship with the host but any instability in these microbiomes leads to several GI pathologies, including IBS [6]. It has also been reported that patients with severe IBS have a greater magnitude of gut microbiota dysbiosis [3]. It is difficult to study the microbiological profile of the diverse microbiome in the gut with classical culture methods. However, recent technologies such as metagenomics and metatranscriptomics have shed light on the study of gut microbiota [2]. A one-year population-based prospective study concluded that IBS had a bidirectional relationship with psychiatric conditions such as anxiety and depression, indicating IBS as a disorder of the brain-gut interaction [8,9].

This review paper discusses the possible mechanisms and role of intestinal microbiota, in particular in the pathophysiology of IBS and also its future implications for the prevention of the disease. 

## Review

Method

We used PubMed, PubMed Central (PMC), and Medline as our primary databases to search for articles using keywords and medical subject heading (MeSH) keywords, as shown in Table 1 and Table 2 on April 30, 2022. The keywords used were Irritable Bowel Syndrome, IBS, Gut Microbiota, Gut-Brain axis, Visceral Hypersensitivity, and the MeSH keywords used were "( "Irritable Bowel Syndrome/diagnosis"[Majr] "Irritable Bowel Syndrome/etiology"[Majr] "Irritable Bowel Syndrome/microbiology"[Majr], Gastrointestinal Microbiome/physiology"[Majr]. We used boolean operators like “AND” and “OR” to combine the keywords and MeSH keywords to search for relevant articles. 

**Table 1:** Keywords IBS: Irritable bowel syndrome

KEYWORDS	DATABASE	INITIAL SEARCH WITHOUT APPLYING INCLUSION/ EXCLUSION CRITERIA	AFTER APPLICATION OF INCLUSION/ EXCLUSION CRITERIA
Irritable Bowel Syndrome	PUBMED	16,310	1,483
IBS	PUBMED	17,554	2,307
Gut Microbiota	PUBMED	53,817	12,039
Gut Brain Axis	PUBMED	4,619	967
Visceral Hypersensitivity	PUBMED	2,592	157

**Table 2:** MeSH Keywords MeSH: Medical subject headings

MeSH KEYWORDS	INITIAL SEARCH WITHOUT APPLYING INCLUSION/ EXCLUSION CRITERIA	AFTER THE APPLICATION OF INCLUSION/EXCLUSION CRITERIA
( "Irritable Bowel Syndrome/diagnosis"[Majr] OR "Irritable Bowel Syndrome/etiology"[Majr] OR "Irritable Bowel Syndrome/microbiology"[Majr] OR "Irritable Bowel Syndrome/pathology"[Majr] OR "Irritable Bowel Syndrome/physiology"[Majr] OR "Irritable Bowel Syndrome/physiopathology"[Majr] )	3,683	446
( "Gastrointestinal Microbiome/etiology"[Majr] OR "Gastrointestinal Microbiome/genetics"[Majr] OR "Gastrointestinal Microbiome/immunology"[Majr] OR "Gastrointestinal Microbiome/physiology"[Majr] )	7,657	2,915

In our review article, we included the articles that were published in the last five years (2017-2022) that were related to human studies irrespective of age, race, gender, and geography. We selected papers that were published in the English language only. We included all types of studies, including observational studies, clinical trials, cross-sectional studies, systematic reviews, and traditional reviews that were available as full-text articles. Other articles which were not in English, had duplicate studies, were published before 2017, or the studies that included animals were excluded from this review.

Results

**Figure 1:**
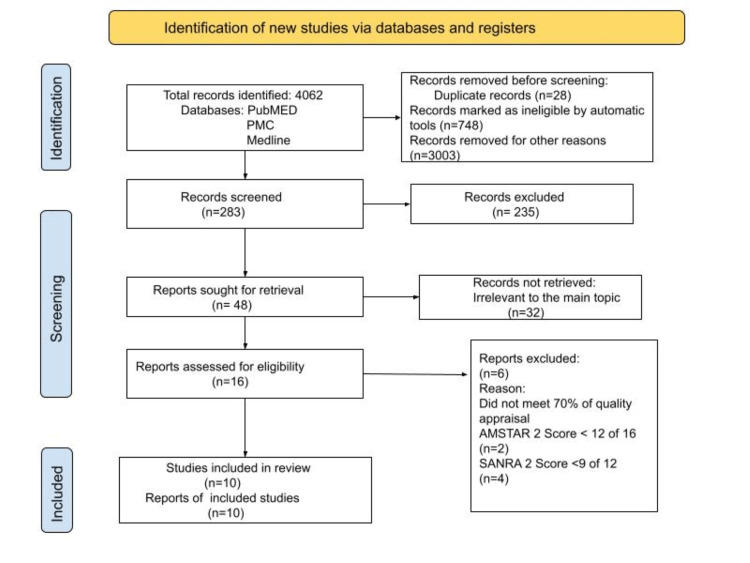
PRISMA Chart PRISMA: Preferred reporting items for systematic reviews and meta-analysis

This study follows the rules listed within the Preferred Reporting Items for Systematic Reviews and Meta-Analyses (PRISMA) checklist of 2020, which has been shown in Figure 1. We were able to retrieve a total of 4062 studies in the initial search from the electronic databases after combining the keywords and MeSH keywords. Twenty-eight duplicate articles were removed using Endnote. A total of 283 records were screened out of which 48 papers were sought for retrieval. The abstract and full-text articles were reviewed, and the number of relevant articles was reduced to 16 and assessed for eligibility. Finally, 10 articles were selected and included in this paper after they met at least a score of 70% in the quality assessment. Two independent authors assessed the studies using Newcastle-Ottawa Scale (NOS) for observational studies, the Assessment of Multiple Systematic Reviews (AMSTAR) 2 checklist for systematic reviews, the scale for the assessment of narrative review articles (SANRA) checklist for literature review articles, and the Cochrane risk-of-bias tool for randomized controlled trials. We have included five review articles, two case-control studies, two randomized controlled trials, and one cross-sectional study in this paper, and the summary and characteristics of the studies have been described in Table 3.

 

**Table 3:** Summary of the studies that depict the role of gut microbiota in the pathogenesis and therapy of the Irritable Bowel Syndrome.

SN	Author	Year of Publication	Type of Study	Objective of the Study	Conclusion/ Result of the Study
1	Harris et. al [3]	2017	Review	To provide an overview of the role of gut microbiota in IBS.	The modulation of gut microbiota with dietary modifications, prebiotics, probiotics, synbiotics, and non-systemic antibiotics are efficacious in treating IBS. However, further clinical trials are necessary to identify species and strain-specific effects.
2	Bhattarai et.al [6]	2017	Review	To study the effect of various factors such as host genetics, stress, diet, and antibiotics in the composition of gut microbiota in IBS.	The gastrointestinal motility and sensation, gut-brain axis, immune activation, and intestinal barrier function are influenced by the gut microbiome, and these are involved as the underlying mechanism of IBS. Longitudinal studies are encouraged to establish the causality of IBS and develop target-specific therapies.
3	Moser et. al [10]	2017	Review	To discuss the strong correlation of intestinal microbiome-gut-brain axis with IBS.	There is a bidirectional link between the intestine and the nervous system. IBS can result from an altered gut microbiome related to psychological stress.
4	Peter et. al [11]	2018	Randomized controlled trial	To assess the correlation of microbiome and psychological distress in IBS patients.	Out of the 48 IBS patients, 65% had psychological distress, 31% had anxiety and 21% had depression, and showed an association with microbial beta diversity. Lachnospiraceae abundance was negatively related to depression. Elevated Bacteroidaceae were seen in patients with anxiety. Patients with psychological distress were found to have a signature of 148 unclassified species.
5	Salem et. al [4]	2018	Review	To understand gut microbiota as a significant pathogenic factor in IBS.	The alteration in the gut microbiota leads to IBS symptoms and is also a contributing factor to CNS-related comorbidities in IBS.
6.	Wei et. al [12]	2020	Case-control study	To investigate the fecal bile acid profile of IBS-D patients and healthy controls to explore the relation between bile acids (BAs) and clinical characteristics as well as the gut microbiota of IBS-D patients.	The abundance of genera Ruminococcaceae was significantly reduced in the 55 IBS-D patients. Also, the altered metabolism of bile acids (increase in primary BAs and decrease in secondary BAs) in these patients correlated with IBS symptoms such as diarrhea and visceral hypersensitivity.
7	Liu et. al [13]	2020	Case-Control study	To determine the microbial patterns in correlation with anxiety and depression in IBS-D patients.	The fecal microbiota study of 70 IBS-D patients showed depleted *Faecalibacterium, Eubacterium* group, *Subdoligranulum*, and increased* Prevotella*. Dialister showed a negative association with IBS severity, anxiety, and depression level. IBS severity was also negatively associated with Roseburia.
8	Mishima et. al [2]	2020	Review	To discuss the molecular mechanisms in the pathogenesis of IBS.	Intestinal dysbiosis and microbiome-derived neurotransmitters, compounds, metabolites, neuroendocrine factors, and enzymes were found to be involved in the pathogenesis of IBS.
9	Baranduzi et. al [14]	2021	Cross-sectional Study	To study the link between food components and gut microbiota patterns between IBS patients and healthy controls (HC).	80 IBS patients and 21 HC were recruited for the study and 16S rRNA Illumina sequencing of the fecal samples showed higher alpha diversity indices and altered gut microbiota in IBS patients who consumed caffeine of more than 400 mg/d.
10	Yang et. al [15]	2021	Randomized Control Trial	To describe the effects of *Lactobacillus Plantarum *CCFM1143 as a probiotic therapy for chronic diarrhea.	*Lactobacillus Plantarum* CCFM1143 was seen to alleviate the bowel frequency by inhibiting the increase in IL-6 and regulating the gut microbiota. 28 patients who were given the probiotic informed an improvement in their clinical symptoms and quality of life as compared to the 27 patients who were in the placebo group.

Discussion

Pathophysiology of IBS

IBS is recognized as a heterogeneous disorder, and the various factors implicated in its pathophysiology include host as well as environmental factors, intestinal dysmotility, increased intestinal permeability, mucosal immune dysfunction, alteration in the brain-gut interaction, enteric infections, visceral hypersensitivity, and psychological disorder [16]. Table 4 is a summary of the proposed factors as the cause of IBS and their role in the pathophysiology of the disease process of IBS.

**Table 4:** Table showing the proposed factors as the cause of IBS and their role in the pathophysiology of the disease process of IBS. IBS: Irritable bowel syndrome

Factor implicated as the cause of IBS	Description
Genetic	About one-third of IBS patients have a positive family history. Twin studies have shown higher concordance of IBS in monozygotic than dizygotic twins. Mutation and gene polymorphism of serotonin receptors such as Serotonin reuptake receptor (SERT) and sucrose isomaltase (SCN5A) have been reported in IBS [17,18].
Diet	Food intolerance to fermentable oligosaccharides, disaccharides, monosaccharides, and polyols (FODMAPs) diet causes osmotic hypertension in the small intestine leading to excessive colonic gas production and other functional GI symptoms [19,14].
Gastrointestinal Factors	The gastrointestinal factors include bile acid malabsorption, mucosal inflammation, increase in intestinal permeability, imbalance in the gut microbiome, and enteric infections. It is seen that the risk for IBS increases six-fold post-infection. Some of the common organisms that can lead to IBS include* Norwalk* virus, *Escherichia coli, Clostridium difficile*, *Campylobacter jejuni*, and *Giardia intestinalis* [3,17].
Visceral Hypersensitivity	Visceral hypersensitivity is known as the keystone for the pathogenesis of IBS. It is the increased perception of luminal stimuli due to the increased sensitization of visceral pain pathways at peripheral, spinal, and supraspinal levels. Adult and pediatric studies have demonstrated decreased rectal sensory threshold for pain in IBS and functional abdominal pain [18,20].
Psychological	Psychological conditions are prevalent in about 94% of patients with IBS [19]. Baseline depression or anxiety has been associated as a risk factor for developing new-onset IBS according to a 12- year cohort study. Also, patients with functional IBS risk developing depression or anxiety, thus showing a bidirectional association between the two disorders. This also implies the brain-to-gut and gut-to-brain pathophysiological association [21]. Another study done on military personnel showed that post-traumatic stress disorder (PTSD) and other psychosocial stress led to an increased risk of post-infectious IBS [22].

Gut-Microbiota in the Pathophysiology of IBS

Around 100 trillion microorganisms belonging to hundreds of different species colonize the human gastrointestinal tract [23]. This diverse complex community of the gastrointestinal microbiota resides in the gut maintaining a mutual symbiosis with the host [4]. The host accommodates the microbes by providing a nutritious and hospitable environment, and in return, the microbiota plays a key role in many of the physiological and metabolic processes such as maintaining immune homeostasis, intestinal epithelial barrier, fermentation of undigested carbohydrates, and also provides protection against the colonization of pathogens [24,25]. The cecum and proximal colon hosts the highest density of microbiota, and the large, as well as small intestines, have similar biomass of microbiota. The composition of microbiota varies a lot with aging. Normally, strict anaerobes such as* Bacteroidetes* and *Firmicutes* dominate the healthy colon and usually remain stable over the years [23,25]. Overall, the gut microbiota helps in the well-being and maintenance of a healthy ecosystem in the host [2].

However, the consistency of gut-microbiota depends largely on the diet, ingested drugs, the intestinal mucosa, and the composition of the microbiota itself. Microbial dysbiosis occurs when there is an imbalance or alteration in the ratio of the microorganisms caused by oxidative stress, bacteriophage induction, and the secretion of bacterial toxins. It has been implicated as a cause of various inflammatory, autoimmune, metabolic, and even neurological disorders. Dysbiosis is associated with a number of gastrointestinal pathologies, including IBS. It is even related to the promotion of colorectal cancer, and it is also known as one of the hallmarks of ulcerative colitis and Crohn's disease [26]. 

The rapid progression of recent microbiome research using advanced microbiological technologies has shed light on dysbiosis related to the pathophysiology of IBS. Approximately, 10% of the IBS cases have been reported after an episode of gastroenteritis leading to post-infectious IBS. The symptom severity of IBS has also been associated negatively with the density of the microbiome [6]. Despite the variable findings in different studies, most studies have concluded that IBS patients have a reduction in bacterial diversity and an increase in the temporal instability of the microbiota [4]. In a study conducted between IBS patients and the healthy control group, a lower number of* Bacteroidetes* and a higher number of* Firmicutes* were found in IBS patients.* Verrucomicrobia, Proteobacteria, Actinobacteria,* and* Ruminococcus *were also found in higher abundance in IBS patients [14]. On the contrary, another report showed a lower number of* Bacteroidetes *in IBS patients. Lower concentrations of aerobic bacteria (1.4*10^7 colony-forming units [CFU] g/feces) were found in patients with IBS-D when compared to the healthy control group (8.4*10^8 CFUs/g feces) [27]. In a meta-analysis, IBS patients were found to have higher levels of fecal* Escherichia coli* and *Enterobacter* and lower levels of fecal *Lactobacillus *and* Bifidobacterium* [2]. 

*Clostridium difficile, Escherichia coli, Mycobacterium avium subspecies Paratuberculosis, Campylobacter concisus, Campylobacter jejuni, Chlamydia trachomatis, Helicobacter pylori, Pseudomonas aeruginosa, Salmonella spp, Shigella spp, Giardia lamblia, *and viruses, particularly *Noroviruses* are the some of the most common pathogens involved in the exacerbation of IBS [28]. *Campylobacter *infections disrupt the intestinal barrier leading to cell death and increasing gut permeability. Furthermore, these infections lead to the increment of various immune-related cells such as macrophages, mast cells, T-lymphocytes, and pro-inflammatory cytokines (TNF-α, IFN-γ, IL-3, IL-4, IL-5, and IL-6) which has a notable effect on the vascular permeability, gastrointestinal motility, secretion, and pain signaling [17]. 

**Figure 2:**
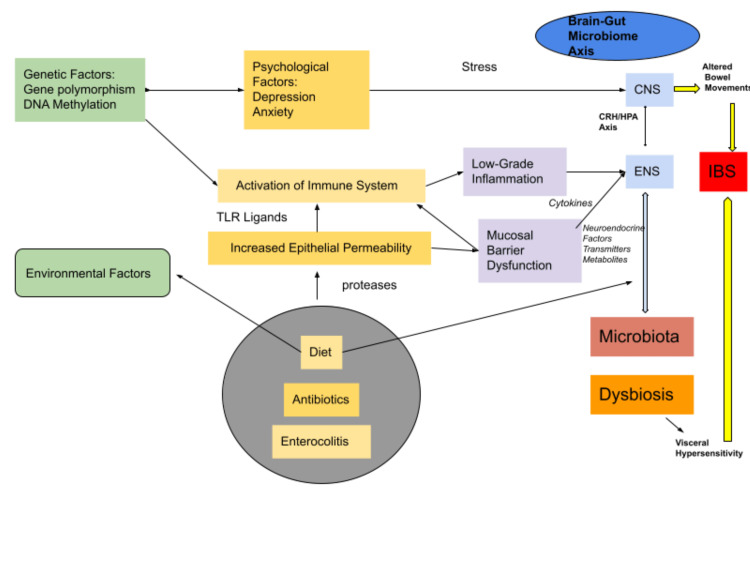
Figure showing the relation of gut microbiota in IBS. Adapted from Mishima Y et al. [2] IBS: Irritable bowel syndrome; CNS: Central nervous system; ENS: Enteric nervous system; TLR: Toll-like receptor; CRH/HPA: Corticotropin-releasing hormone/ Hypothalamic-pituitary-adrenal axis

The pathogenesis of IBS involves the interaction of gut microbiota with the host to produce several metabolic substances such as bile acids, neurotransmitters, short-chain fatty acids, and other signaling factors. In the human intestine microorganisms such as *Listeria monocytogenes, B. vulgatus, Lactobacillus, Clostridium perfringens, Bifidobacterium, *and* Bacteroides fragilis* are involved in the production of secondary bile acids. Also, alteration in the concentration of bile acids causes cytotoxicity leading to apoptosis, necrosis, DNA damage, and functional gastrointestinal disorders [27]. Wei Wei et al. conducted a study on 55 patients with IBS-D and investigated the role of bile acids in the pathogenesis of the disease. They concluded that the IBS-D patients had an increase in the primary bile acids and a decrease in the secondary bile acid in the feces, which correlated with the reduction in the *Ruminococcaceae *family [12]. Figure 2 is a depiction of gut microbiota and its relation with other factors such as diet, antibiotics, and environmental and psychological factors that play a role in the pathogenesis of IBS and its symptoms. 

The Brain-Gut Microbiome Axis

In the past few decades, numerous studies have highlighted gut-microbiota as a key regulator in the brain-gut microbiome axis, and studies have shown the activation of important neuronal pathways in IBS pathways [9]. The brain-gut microbiome axis is formed with the complex communication through neuronal, endocrine, and immune signaling between microbiota and the Central Nervous System (CNS). The gut microbiota is influenced by CNS with the stress-mediator-induced virulence gene expression and the control of gastrointestinal functions, including motility and immune modulation through the Autonomic Nervous System (ANS). Additionally, the Enteric Nervous System (ENS) is also involved in the alteration in gastrointestinal functions causing changes in the composition of the microbiota [8]. A study done using fMRI observed functional and structural changes in the right hippocampus in patients with a large abundance of *Pervotella* as compared to patients who had a large abundance of *Bacteroides*. Another study showed that probiotic *B. longum *caused a reduction of responses to negative emotional stimuli in the amygdala and fronto-limbic regions [9]. 

The gut-microbiome-brain axis is regulated via the production of metabolites such as short-chain fatty acids (SCFAs), serotonin, tryptophan, and tryptamine. SCFAs are responsible for the promotion of inflammatory cytokine production and recruitment of T-cells and neutrophils, causing neuroinflammation. The gut microbiota produces Butyrate, an SCFA which promotes memory and neuronal plasticity by inhibiting histone deacetylases. They stimulate the enteroendocrine cells of the gut epithelium, which diffuses through the lamina propria and affects the ENS and vagal innervation. Serotonin is involved in functions such as gastrointestinal secretion and peristalsis, vasoconstriction, behavior, and neurological functions. In comparison to healthy individuals, IBS patients have been reported to have lower mucosal and higher systemic concentrations of 5-HT (5-hydroxytryptamine) and Kynurenic acid (KYNA). This was correlated with the association of the diverse microbiota found in IBS and the psychological disorders, which were evaluated with the Hospital Anxiety and Depression Scale and the Symptom Checklist-90. *Escherichia coli, Achromobacter liquefaciens, *and* Paracolobacturm coliforme* produces pyruvate by breaking down tryptophan into indole with the help of the enzyme tryptophanase. Large amounts of pyruvate are toxic to the gut epithelium [29-32].

The gut microbiota is also involved in the maturation of the hypothalamic-pituitary-adrenal axis (HPA-axis). IBS is known as a stress disorder, and gut dysbiosis has been related to the involvement of HPA-axis and ANS, maladaptive coping, comorbidity of anxiety and depression, and changes in neuronal pain processing [10]. Psychological stress and depression have shown a correlation with dysbiosis leading to IBS. An increase in *Escherichia coli *and *Pseudomonas *and reduction in *Lactobacilli *in patients with chronic psychological stress; and an increase in *Enterobacteriaceae* in patients with depression have been noted [6]. Additionally, an increase in *Streptococcus, Klebsiella, Prevotella, *and *Clostridium XI* and altered *Firmicutes* to *Bacteroidetes* ratio was noticed in patients with the major depressive disorder [10]. *Aspergillus fumigatus, Candida albicans, *and *Saccharomyces cerevisiae* were found to increase the production of cytokines such as IL-6 in the intestinal mucosa. These cytokines are associated with the activation of the HPA axis and the increase in cortisol which has been linked to the potential for the development of new-onset depression in IBS patients [33].

Henceforth, a bidirectional relationship has been established between the gut microbiome and the nervous system that alters the course of IBS. Reports have shown that 75% of IBS patients had psycho-social comorbidity, with approximately (30%-50%) suffering from anxiety and hopelessness, about 30% presenting with mood disorders, and (15%-30%) experiencing suicidal thoughts [17].

Therapeutic Use of Microbiota-Directed Therapies in IBS

The potential of dietary manipulation of gut microbiota and the use of probiotics, prebiotics, and synbiotics in the treatment of IBS has been studied in recent years and shown promising results, as shown in Table 5 [34].

**Table 5:** Gut microbiota-related IBS therapies. Adapted from Janeiro BKR et al. [34] IBS: Irritable bowel syndrome; FODMAP: Fermentable oligosaccharides, disaccharides, monosaccharides, and polyols

IBS therapies	Implicated Microbiota
Probiotics	It reduces the number of competing pathogens by both productions of antimicrobial substances and interference in intestinal mucosal adhesion (example: *Lactobacillus sp.* and *Bifidobacterium sp* ).
Prebiotics	Classified as disaccharides, such as lactulose, and oligosaccharides. It promotes the growth of *Bacteroides*, *lactobacilli, *and especially *Bifidobacterium.*
Synbiotics	A combination of* L. acidophilus, L. helveticus,* and *Bifidobacterium* species in vitamin and phyloextract-enriched medium for 12 weeks in IBS patients was found to be effective in symptom control
Non-absorbable Antibiotics	Rifaximin decreases bloating, abdominal pain, abdominal distension, and flatulence in IBS patients.
Dietary Modification	A Low FODMAP diet is recommended.
Future considerations and possible treatments	Genetic engineering of bacteria and microbiota manipulation, bacteriophage therapy, fecal transplantation, postbiotics, drug-mediated manipulation of the gut microbiome, and new probiotics can be implicated in the future.

Probiotics are known to modulate immune functions, enhance the intestinal mucosal barrier and reduce inflammation in IBS patients. They help to increase short-chain fatty acids, which facilitates the colonization of beneficial strains* Lactobacillus*, *Bifidobacterium,* and a few probiotic bacterias also decrease colonic hypersensitivity by elevating the μ-opioid and cannabinoid receptors expression [33]. The combination of *L.acidophilus Rosell-*52 and *B. Longum*-175 as a prebiotic has been proven to be successful in relieving stress-related gastrointestinal symptoms [9]. In a double-blinded randomized control trial conducted in 50 IBS-D patients supplementation of probiotics using microorganisms *Lactobacillus acidophilus, L. plantarum, L. rhamnosus, Bifidobacterium breve, B. lactis, B. longum,* and* Streptococcus thermophilus *resulted in significant improvement of depressive symptoms which were most probably modulated by the gut-axis [17].

The foods that are resistant to enzymatic and chemical digestion and promote the proliferation of healthy gut microbiota are known as prebiotics. IBS patients treated with four weeks of prebiotic have shown improvement in symptoms such as bloating, flatulence, and stool consistency. Also, in a study of IBS patients, *Bifidobacterium* colonization was seen to increase after the intake of the prebiotic trans-galactooligosaccharide mixture [33,34].

Dietary modifications such as low fermentable oligosaccharides, disaccharides, monosaccharides, and polyols (FODMAPs) diets are being recommended for IBS treatment. The patients with IBS have reported a significant reduction in pain and bloating with a low FODMAP diet. Decreases in the levels of fecal *Actinobacteria, Bifidobacterium, *and* Faecalibacterium*, total SCFAs, and n-butyric acid along with serum proinflammatory IL-6 and IL-8 have also been reported in several studies in IBS patients on the low FODMAP diet. Fecal microbiota transplantation (FMT) has also been considered for the restoration of the healthy gut-microbiome and treatment of IBS. A randomized controlled trial in IBS-D and IBS-M showed symptom relief at three months from active treatment with FMT but not at 12 months. The clinical studies are insufficient regarding FMT, and further affirmation is required through other larger clinical trials [8,34].

Limitations

This review paper has several limitations as we restricted our search to select papers that were published within the last five years. We included papers with free full-text articles published in the English language only. Also, we selected papers that included human studies which might have missed out on papers with relevant information regarding this topic. We also found variation and conflict in data and the need for papers with longer follow-ups and a larger population. Furthermore, data supporting microbiome-related therapies were scarce. Therefore, the study of gut microbiomes is vast and requires further longitudinal studies and clinical trials to provide more evidence on the pathogenesis of IBS.

## Conclusions

In conclusion, the gut microbiome plays a substantial role in the pathophysiology of IBS. Gut dysbiosis was found to be common in IBS patients, and the production of various metabolites such as bile acids, short-chain fatty acids, and neurotransmitters direct the symptoms of IBS. The intricate relationship of the gut-microbiome-brain axis has shown a bidirectional association of IBS with psychosocial disorders. Also, the growing and considerable evidence of gut microbiota in the pathogenesis of IBS has also led to promising research studies on the therapeutic use of microbiome for IBS. Nevertheless, further well-defined longitudinal studies and clinical trials with larger populations and longer follow-up periods are required to establish the role of the gut microbiome in the disease process and the management of IBS.
